# An e-Learning Intervention for Professionals to Promote Family-Centered Cancer Care When a Significant Caregiver for Children Is at End of Life: Mixed Methods Evaluation Study

**DOI:** 10.2196/65619

**Published:** 2024-12-10

**Authors:** Cherith Jane Semple, Carla O'Neill, Sarah Sheehan, Tanya McCance, Amanda Drury, Jeffrey R Hanna

**Affiliations:** 1 South Eastern Health & Social Care Trust Ulster University Belfast United Kingdom; 2 University College Dublin Dublin Ireland; 3 Ulster University Belfast United Kingdom; 4 Dublin City University Dublin Ireland

**Keywords:** e-learning, intervention evaluation, mixed methods, end-of-life care, educational intervention, professionals, self-efficacy, cancer, family-centered care, cancer care, person-based approach, qualitative, evidence-based, parent, adverse outcome, eHealth

## Abstract

**Background:**

Families are often unsure how best to prepare dependent children for the death of a significant caregiver with a poor cancer prognosis and seek guidance and support from health care teams. Health and social care professionals (hereafter referred to as *professionals*) often lack educational opportunities to gain the desired knowledge, skills, and confidence to provide family-centered supportive cancer care. e-Learning has positively impacted access and reach, improving educational opportunities in health care.

**Objective:**

We aimed to evaluate the acceptability, usability, and effectiveness of an evidence-based, theory-driven e-learning intervention to equip and promote professionals’ self-efficacy to deliver family-centered supportive cancer care when a significant caregiving member for dependent children is at the end of life.

**Methods:**

Guided by the “person-based approach,” a mixed methods outcome evaluation was used. To determine the effect on self-efficacy, participants completed a validated pretest and posttest 12-item self-efficacy survey. The use of one-on-one, remote semistructured interviews and single-item questions determined the usability by professionals of the e-learning intervention and the acceptability of perceived learning in clinical practice. To generate enhanced insights, quantitative and qualitative data were integrated through a 4-stage, modified pillar integration process.

**Results:**

Overall, 158 participants completed the pretest survey for the e-learning resource, with 99 (62.7%) completing the posttest survey. Semistructured interviews were conducted with 12 professionals at least 1 month after the intervention. Findings highlighted a statistically significant improvement in posttest self-efficacy (99/158, 62.7%; *P*<.001). Usability of the e-learning intervention was positive, with participants reporting that it was clear and organized (mean 4.84, SD 0.373), the layout was appealing (mean 4.71, SD 0.539), the language was easy to understand (mean 4.71, SD 0.407), and graphics and media were purposeful (mean 4.76, SD 0.495) and engaging (mean 4.67, SD 0.703). Determining acceptability, participants considered that the intervention would positively impact practice (mean 4.60, SD 0.589) and increase knowledge (mean 4.56, SD 0.677), with appropriate practical examples to support learning (mean 4.58, SD 0.610). Following engagement with the e-learning intervention, professionals reported preparedness to deliver supportive adult-professional end-of-life cancer care, when an adult with significant caregiving responsibilities is dying. Findings demonstrated transferable learning to additional contexts, such as other close adult-child relational bonds (grandparents) and to life-limiting conditions.

**Conclusions:**

The systematic and iterative person-based approach optimized the acceptability of a novel e-learning intervention, having the potential to promote family-centered supportive end-of-life cancer care. This accessible e-learning intervention makes an important contribution to the recognized global gap of educational interventions in this field. Equipping professionals with family-centered supportive end-of-life care improves self-efficacy and preparedness to engage in challenging conversations, with the potential to promote better outcomes for affected adults and children and mediate adverse outcomes for adults and children before and after bereavement.

## Introduction

A recent systematic review indicated that a substantial proportion (14%-25%) of people diagnosed with cancer are parents or have significant caregiving responsibilities for dependent children (aged <18 years) [1]. Having cancer alongside significant caregiving responsibilities for children presents unique challenges, contributing significantly to higher levels of depression and anxiety [2]. Strikingly, 1 in 5 dependent children who have a parent with cancer experience the death of their parent from cancer [3].

Many families are often unsure how best to prepare children for the end-of-life experience of a significant caregiver who has a poor cancer prognosis [4]. Often, adults in this situation not only experience higher levels of psychological distress but also are at a greater risk of family dysfunction [3]. In recent years, there has been an increased focus on communication challenges within families and the provision of supportive programs [5,6]. Nonetheless, the needs of families frequently remain unmet and efforts to address the psychosocial needs of families when a significant adult is at the end of life remain in its infancy [5]. Consequently, many children are unprepared for this expectant and traumatic experience, predisposing them to adverse outcomes before and after bereavement [3,7]. Studies have shown that a child who is unprepared for the death of a significant adult in childhood is associated with an increased risk of mental health problems and threats to emotional well-being, such as anxiety, depression, and a perceived lack of control over what happens in one’s life [7,8]. It is also correlated with increased criminality rates, risk-taking behaviors, behavioral problems, and poor educational attainment [3,7].

Health and social care professionals (hereafter referred to as *professionals*) are well-placed to support families as they prepare children for the death of a significant adult caregiver to cancer [9-11]. Despite this, professionals often avoid significant end-of-life conversations with adults regarding their caregiving responsibilities for children for fear of making a challenging and fraught situation worse [12,13]. Professionals have repeatedly highlighted a need for training to equip themselves with the skills, knowledge, and confidence to provide meaningful family-centered cancer care at the end of life [9,12,13]. Despite this, a systematic review conducted by our team identified a paucity of available educational interventions for professionals (n=2) and an imminent need for robust educational interventions to be developed [5].

Given the avid need for training, our team designed and evaluated a face-to-face, evidence-based, and theory-driven educational intervention for professionals to support adult-child end-of-life communication and management [14]. A mixed methods evaluation demonstrated statistically significant (*P*<.001) improvements in professionals’ perceived self-efficacy after completing the face-to-face educational intervention, with in-depth interviews after the intervention identifying that clinicians had gained new tools and strategies on how to start supportive conversations with adults regarding the children [14]. To progress family-centered end-of-life cancer conversations in routine practice at a global level, there is an inherent need to promote the accessibility and availability of the provision of such education [14,15].

There is evidence to suggest that greater flexibility and access to digital education for professionals is transforming the delivery of health care education [16]. It also positively impacts promoting person-centered outcomes, with patients and carers reporting greater satisfaction, empowerment, and inclusion in their physical and psychological health needs [17-19]. It has been suggested that the *success* of e-learning interventions in promoting positive behavior change is associated with the interventions being cocreated with end users. This ensures that the content, features, and navigational aspects of the resource are relevant, applicable, motivating, enjoyable, and informative [20-22]. These principles align with the person-based approach to developing e-learning interventions [23].

Guided by the person-based approach, the research team sought to adapt the face-to-face educational intervention [14] to a stand-alone, self-directed e-learning intervention for professionals. To support this process, key behavioral issues, needs, and challenges that the e-learning intervention must address were outlined [23]. This consisted of the development of a logic model that was underpinned by the family resilience theory [24] and social cognitive theory [25]. In addition, guiding principles were developed, comprising design objectives and distinctive features of the e-learning intervention that were necessary to achieve the project objectives and logic model [26]. The research team collaborated with an expert group and a team of learning technologists to refine the content of the face-to-face educational intervention [14] and identified appropriate and relevant components for the e-learning intervention (the content and logic model has been published in detail elsewhere) [26]. The e-learning intervention prototype was developed using the authoring tool RISE360 (Articulate). Alongside appropriate visuals and icons, interactive elements were incorporated to the e-learning intervention prototype such as flip-card activities, expandable icons, and reflective activities [26].

The e-learning intervention prototype was optimized with end users to ensure it was appropriate, relevant, and applicable [27]. An iterative approach involving 3 cycles of data collection and analysis was used to enact changes to the e-learning intervention with professionals and patient and public involvement representatives [26]. In line with the person-based approach, there is a need to determine the effectiveness, usability, and acceptability of the e-learning intervention [16,27,28].

Using the person-based approach, the aim of this study is to evaluate the acceptability, usability, effectiveness, and outcomes of an e-learning intervention developed to equip professionals to deliver family-centered supportive cancer care when an adult with significant caregiving responsibilities for children is at the end of life with cancer. The objectives of the study are as follows:

1. Explore professionals’ acceptability and usability of engaging with an e-learning intervention on how best to support adults at the end of life with cancer who have significant caregiving responsibilities for dependent children.
2. Determine the effectiveness of an e-learning intervention on professionals’ perceived self-efficacy to provide family-centered end-of-life cancer care in routine practice.
3. Explore professionals’ experience and perceived learning from the e-learning intervention in clinical practice.

## Methods

### Overview

To guide the evaluation process, this project focuses on levels 1 and 2 (reactions and learning) of the 4 levels of evaluation in Kirkpatrick’s model [29]. Using a mixed methods approach, quantitative pretest and posttest surveys were completed before and after participants completed the e-learning intervention. Qualitative interviews were conducted at least 4 weeks after completion of the e-learning intervention to further explore users’ experience of and perceived impact of the e-learning intervention on professionals’ practice. The main purpose of adopting a mixed methods design was to enable both complementarity and expansion of data, with both quantitative and qualitative components given equal weighting for this applied research study of a complex issue.

### Setting

Between October 2023 and June 2024, individuals were invited through a study advertisement to evaluate the e-learning intervention if they were a health and social care professional involved in the care of families impacted by cancer at the end of life. There was no limitation placed on geographic location, health or social care context, or health care provider. Participants were informed about the resource evaluation through social media posts on X (formerly Twitter), hospice organizations, flyers that were distributed at relevant national and international cancer and palliative care conferences and events and the research team’s professional networks.

### Sampling

A minimum sample of 34 was required (power calculation in G*power^,^ based on repeated measures 2-tailed *t* test using a power of 0.8 and medium effect size of 0.05, with a Cronbach α value of 0.05). A planned sample size of 50 was selected to allow for missing data at follow up [30].

Eligibility criteria included registered health or social care professionals involved in end-of-life care of families impacted by cancer, who consented to the study with an ability to understand and converse in English. Trainee health and social care professionals were excluded from participation.

### Procedure

The study advertisement indicated that individuals could access the e-learning intervention by registering for a free account on the All-Ireland Institute of Hospice and Palliative Care’s Palliative Hub Learning Platform. Eligible participants could then access the participant information sheet and consent form that were embedded in a Qualtrics survey on a separate URL link. The participant information sheet provided individuals with explanations as to why the study was being conducted, what participation would include, the risks involved, and whom to contact if they had any questions regarding the study. Once interested and willing professionals completed the consent form, the pretest survey was automatically loaded within the Qualtrics link on the learning platform page. Professionals could only access the pretest survey if they completed the consent form. An honor-based system was in place where participants could access the e-learning intervention without completing a pretest survey.

### Data Collection

#### Pretest Survey

Participants completed the Self-Efficacy Questionnaire (SE-12) for measuring clinical communication skills on a Likert scale ranging from 0 (very uncertain) to 10 (very certain) for 12 questions [31]*.* The maximum score was 120, which reflects high self-efficacy [31]. Of note, the framing of questions in the SE-12 was applied to conversations with adults at the end of life with cancer regarding the children. Multimedia Appendix 1 presents the modified version of the SE-12 scale used in this study. Appropriate demographic questions were included to capture details about the convenience sample. Participants were asked to create a unique code to match each of their pretest and posttest surveys with their first initial and date of birth in the format “xddmmyy.”

#### Posttest Survey

The posttest survey included the same version of the SE-12 scale as the pretest survey [31]. Alongside this, participants completed single-item questions to evaluate the usefulness and relevance of the e-learning interventions on a 5-point Likert scale ranging from 1 (strongly disagree) to 5 (strongly agree). The same demographic questions were included in the posttest survey to capture details about individuals who may have only completed the posttest survey.

#### Qualitative Interviews

A total of 29 email addresses were provided by participants who completed the posttest survey. These individuals were emailed at least 4 weeks after completing the posttest survey and were provided with a participant information sheet and consent form from the third author (SS). A follow-up invitation was sent to individuals 2 weeks after the initial invitation to aid participation.

Semistructured interviews were conducted between February and June 2024. A topic guide was developed by the research team, guided by Kirkpatrick’s model of evaluation [29] and other relevant literature (see Textbox 1 for a sample of topics). Interviews were completed when no further categories emerged from the data. Interviews were conducted on Zoom, were audio-recorded, and lasted between 12.5 and 35.47 (mean average 19.96) minutes. Interviews were conducted by the third author (SS) who had no prior relationships with the participants. Recruitment was discontinued following the completion of 12 interviews as no new main themes were being identified.

**Sample of topics explored during the qualitative interviews.**

- Explore professional background, location, and clinical experience.
- Explore professional’s motivations for completing the e-learning intervention.
- Explore the professional’s experience of the e-learning intervention.
- Explore the professional’s perceived learning from the e-learning intervention.
- Explore the professional’s perceived impact of the e-learning intervention on practice.
- Explore the professional’s perception of training needs.
- Anything else that is relevant.

### Data Analysis

#### Overview

The integration of the quantitative and qualitative data was achieved through a modified pillar integration process [32-34]. The process facilitates integration through 4 stages: listing, matching, checking, and pillar building [32]. This process allows meta-inferences about the data to be generated to enhance insights gained relating to the impact of the e-learning intervention [34].

#### Quantitative Analysis

Close-ended questions from the pretest and posttest surveys were analyzed using SPSS (version 29; IBM Corp). Descriptive statistics were used to analyze demographic and single-item questions. A 1-way ANOVA was used to analyze the scores of self-efficacy in the pretest and posttest surveys.

#### Qualitative Analysis

Open-ended questions from the posttest questionnaire and semistructured interviews were analyzed using reflexive thematic analysis [35-37]. Initial coding, generation of initial themes, development and review of themes, and the definition and naming of themes were conducted. Transcripts were transcribed verbatim, and these transcripts were verified by SS. Then, SS coded the data by marking similar words and phrases within the transcripts. This process was managed using NVIVO (version 1.7). Codes were reviewed by CON. Following this, both SS and CON identified where the codes merged as themes. Themes were discussed and refined through critical dialogue with all coauthors.

#### Integration of Quantitative and Qualitative Data

Data from the quantitative and qualitative data were arranged in 2 columns. A description of the interpretation of these findings were listed alongside each data item (ie, listing). Relational quantitative and qualitative data were then aligned with each other in the joint display (ie, matching). These matches were then reviewed and refined independently by all research team members to verify that the matched data represent appropriately matched points of integration (ie, checking). Differences were resolved through critical dialogue as a team. The insights generated from the process were conceptualized as pillars that formed the central column of the joint display (ie, pillar building themes column).

### Ethical Considerations

Participants were informed that all information shared would be confidential. Unique codes were used to match the pretest and posttest survey data. Transcripts were anonymized using unique codes. Data were stored on the University College Dublin OneDrive with password protection and are accessible only by the research team. Data protection procedures are in place to destroy all data in accordance with the General Data Protection Regulation and the Data Protection Act 2018. Ethics approval was obtained from Ulster University (FCNUR-23-002) and University College Dublin (LS-22-65).

## Results

### Overview

Overall, 234 individuals registered to access the course. A total of 158 (67.5%) participants completed the pretest survey, and of those completing the pretest survey 99 (62.7%) participants completed the posttest survey. Of the 158 participants who completed the pretest survey, most were registered nurses (n=100, 63.3%) who were residing in the United Kingdom (n=88, 55.7%) or the Republic of Ireland (n=45, 28.5%). Of the 99 participants who completed the posttest survey, most were registered nurses (n=65, 66%), residing in the United Kingdom (n=59, 60%) or the Republic of Ireland (n=22, 22%).

A total of 12 qualitative interviews were conducted between 4 and 17 weeks after the intervention (m Avg 7.4 weeks). Of these 12 participants, 2 (17%) were medical professionals, 9 (75%) were registered nurses, and 1 (8%) was a health care assistant. These professionals were predominately residing in the United Kingdom (8/12, 67%), and had 22.92 mean average years of clinical experience, with 10.92 mean average years specifically working in cancer care. Sample characteristics are reported in Table 1.

Pillars were generated from an integration of the analysis of the open and closed responses to the pre and posttest surveys and qualitative interviews. A joint display (Multimedia Appendix 2) presents the integrated findings of the quantitative and qualitative data. The integrated analysis generated two pillars: (1) experience of engaging with the e-learning intervention and (2) professionals feeling equipped to have family-centered supportive end-of-life conversations.

**Table 1:** Sample characteristics for all participants (pretest survey, posttest survey, and qualitative interviews).

Characteristics				Participants Values, n (%)
**Pretest survey (n=158)**				
	**Professional role**			
		Registered nurse	100 (63.3)	
		Medical doctor	17 (11.8)	
		Allied health professional	15 (9.5)	
		Social worker	8 (5.1)	
		Counselor	3 (1.9)	
		Support worker (before or after bereavement)	7 (4.4)	
		Health care assistant	6 (3.8)	
		Student nurse	1 (0.6)	
		Psychotherapist	1 (0.6)	
	**Location of participants**			
		Albania	8 (5.1)	
		Australia	1 (0.6)	
		Croatia	1 (0.6)	
		Cyprus	1 (0.6)	
		France	1 (0.6)	
		India	1 (0.6)	
		Ireland	45 (28.5)	
		Malawi	1 (0.6)	
		Malta	1 (0.6)	
		South Africa	1 (0.6)	
		Spain	6 (3.8)	
		Thailand	1 (0.6)	
		Turkey	2 (1.3)	
		United Kingdom	88 (55.7)	
	**Years of professional experience**			
		0-4	15 (9.5)	
		5-9	23 (14.6)	
		10-14	38 (29.7)	
		15-19	15 (11.7)	
		20-24	21 (16.4)	
		25-29	12 (7.6)	
		>30	30 (19)	
		Missing	4 (2.5)	
	**Years of experience working in cancer care**			
		0-4	43 (27.2)	
		5-9	36 (22.8)	
		10-14	27 (17.1)	
		15-19	14 (8.9)	
		20-24	18 (28.4)	
		25-29	7 (4.4)	
		>30	9 (5.7)	
		Missing	4 (2.5)	
	**Previous training**			
		Yes	20 (12.7)	
		Advanced communication skills	6 (3.8)	
		Face-to-face training from team	6 (3.8)	
		Charity organization training	6 (3.8)	
		Postgraduate studies	2 (1.3)	
		No	135 (85.4)	
		Missing	3 (1.9)	
	**Children (aged <18 years) of age at home**			
		Yes	71 (44.9)	
		No	83 (52.5)	
		Missing	4 (2.5)	
**Posttest survey (n=99)**				
	**Professional role**			
		Registered nurse	65 (65.7)	
		Medical doctor	9 (9.1)	
		Allied health professional	6 (6.1)	
		Social worker	8 (8.1)	
		Counselor	3 (3)	
		Support worker (before or after bereavement)	5 (5.1)	
		Health care assistant	1 (1)	
		Missing	2 (2)	
	**Location of participants**			
		Albania	8 (8.1)	
		Australia	1 (1)	
		Croatia	1 (1)	
		France	1 (1)	
		Republic of Ireland	22 (22.2)	
		Malawi	1 (1)	
		South Africa	1 (1)	
		Spain	2 (2)	
		Turkey	1 (1)	
		United Kingdom	59 (59.6)	
		Missing	2 (2)	
	**Years of professional experience**			
		0-4	9 (9.1)	
		5-9	16 (16.2)	
		10-14	22 (22.2)	
		15-19	6 (6.1)	
		20-24	17 (17.2)	
		25-29	7 (7.1)	
		>30	16 (16.2)	
		Missing	6 (6.1)	
	**Years of experience working in cancer care**			
		0-4	24 (24.2)	
		5-9	23 (23.2)	
		10-14	19 (19.2)	
		15-19	8 (8.1)	
		20-24	12 (12.1)	
		25-29	4 (4)	
		>30	3 (3)	
		Missing	6 (6.1)	
	**Previous training**			
		Yes	14 (14.1)	
		No	80 (80.8)	
		Missing	5 (5.1)	
	**Children <18 years of age at home**			
		Yes	41 (41.1)	
		No	53 (53.5)	
		Missing	5 (5.1)	
**Qualitative interviews (n=12)**				
	**Professional role**			
		Registered nurse	9 (75)	
		Palliative care consultant	1 (8.3)	
		Palliative care coordinator	1 (8.3)	
		Health care assistant	1 (8.3)	
	**Location of participants**			
		Australia	1 (8.3)	
		Republic of Ireland	2 (16.7)	
		Spain	1 (8.3)	
		United Kingdom	8 (66.7)	
	**Years of professional experience**			
		0-4	2 (16.7)	
		5-9	0 (0)	
		10-14	1 (8.3)	
		15-19	0 (0)	
		20-24	4 (33.3)	
		25-29	0 (0)	
		>30	5 (41.7)	

### Pillar 1: Experience of Engaging With the e-Learning Intervention

In the posttest survey, participants (97/99, 98%) rated the graphics and media integrated into the resource as being purposeful (mean 4.76, SD 0.495). Similar findings were identified within the open-text posttest responses and interview data, with most participants highlighting the positive and helpful nature of the learning from the videos (Q.1.1.1 and Q.1.1.2; Multimedia Appendix 2):

These short bite size videos like those, they’re excellent, you’re fully engaged right the whole way through and you think oh yes I remember that, that was really good. So that, and also the, I won’t call them actors but the people who participated in the video, you know they were very, very good, spoke very well, spoke very short, concise, gave short concise information which was very beneficial to the actual course.

Interview 02, registered nurse

Some professionals felt there was a need for additional educational video resources roleplaying scenarios of how best to navigate conversations with adults who are emotionally resistant or reluctant to tell their children the reality of the poor cancer prognosis (Q.1.1.3 and Q.1.1.4).

In the posttest survey, participants (97/99, 98%; Multimedia Appendix 2) positively rated the usability of the e-learning intervention, reporting it as easy to use (mean 4.74, SD 0.485; Multimedia Appendix 2), clear, and organized (mean 4.84, SD 0.373; Multimedia Appendix 2), with an appealing layout (mean 4.71, SD 0.539; Multimedia Appendix 2) and with integrated, easy-to-understand language (mean 4.71, SD 0.407; Multimedia Appendix 2). Qualitative data complemented these findings, with professionals positively reporting on the flexibility of being able to complete the e-learning intervention in their own time (Q.2.2.2; Multimedia Appendix 2). Other aspects that were positively reported in the qualitative interviews included the navigational ease of the e-learning intervention (Q.1.2.1, Q.1.2.4, and Q.1.2.5; Multimedia Appendix 2) and the engaging and varying nature of the learning tools such as the interactive elements, videos, and written pieces (Q.1.2.6; Multimedia Appendix 2):

Yeah, like that I thought every segment was well laid out. I think it had a very logical and methodical and the story was well told, you know with that mixture of whether you were clicking on pictures or whether you were watching the videos.

Interview 09, registered nurse

While most professionals highlighted a preference for completing this education as e-learning in the posttest survey, a fifth of the participants considered a face-to-face delivery of the training would have been more appropriate (22/97, 23%; Multimedia Appendix 2). Further explored in the qualitative interviews, participants reported that a blended approach would have enabled an opportunity for further experiential learning in a training environment to aid learning. Participants highlighted the importance of having the opportunity to rehearse difficult family-centered cancer care end-of-life conversations with peers “in a safe space” before integrating within their clinical practice (Q.1.2.3; Multimedia Appendix 2). A participant stated, “...would love a follow up face-to-face, to practice the skill set myself and gain more insight into this topic” (an open-ended response to posttest, registered nurse).

### Pillar 2: Professionals Feeling Equipped to Have Family-Centered Supportive End-of-Life Conversations

Overwhelmingly, participants consistently stated that they had an increased awareness of the importance of engaging in these end-of-life conversations with families (Q.2.1.1, Q.2.1.2, and Q.2.1.3; Multimedia Appendix 2). Alongside this, participants reported that they gained knowledge on how and when to have end-of-life conversations after completing the e-learning intervention, with participants highly rating the single-item “increased knowledge” (97/99, 98%; mean 4.56, SD 0.677; Multimedia Appendix 2). Highlighted in the qualitative data, professionals reflected on acquiring new strategies and tools from the “talking, telling, sharing framework” (Q.2.5.1; Multimedia Appendix 2) on how to open conversations with adults regarding the children, and the appropriate language to provide adults with on how best to share the poor prognosis with the children (Q.2.6.1 and Q.2.6.2; Multimedia Appendix 2):

I thought it was very clear, because again it was something I wasn’t really aware of. Certainly the markers that explained you know the why, where, when, that type of thing. I found that all really useful, because although I won’t necessarily always remember it straight off, if I’m in that situation, I have the tools here I can go back to and remember.

Interview 02, registered nurse

Participants considered the e-learning intervention relevant to their clinical practice (97/99, 98%; mean 4.68, SD 0.550) and were confident that it would positively impact their provision of family-centered cancer care (97/99, 98%; mean 4.60, SD 0.589; Multimedia Appendix 2). Within the qualitative data, professionals highlighted the positive impact that the e-learning intervention would have on their ability to engage in these conversations during their clinical work, with participants describing feeling more equipped to navigate these conversations and better support families (Q.2.2.1, Q.2.2.2, Q.2.2.3, and Q.2.2.4; Multimedia Appendix 2):

It’s made me less fearful, because I didn’t think that was in my role. I didn’t think it was my job, but it is, and now I am very confident to actually do it.

Interview 12, registered nurse

In the posttest survey, 96% (93/97) of participants “agreed” or “strongly agreed” that they would recommend the e-learning intervention to a colleague working in cancer or end-of-life care (Multimedia Appendix 2). This converged with the qualitative findings, with participants highlighting that they had recommended the e-learning intervention to colleagues, clinical managers, and senior staff members as they believed it was relevant and beneficial to their clinical workloads (Q.2.7.1 and Q.2.7.2; Multimedia Appendix 2). Of note, 39.9% (63/158) of the participants in the pretest survey indicated their motivations for completing the e-learning intervention were due to a recommendation from a colleague:

I went to talk with my supervisor. I believe she could share it with the team you know, or with another team. Because I think they would benefit from taking this course.

Interview 08, registered nurse

In the qualitative data, professionals reflected on their learnings and identified its relevance to different family situations, such as grandparents (Q.2.4.3 and Q.2.4.4; Multimedia Appendix 2), other life-limiting conditions, and cultural contexts outside of the one the intervention was developed for (Q.2.4.1 and Q.2.4.2; Multimedia Appendix 2). Some participants considered the e-learning intervention to be especially suitable for nonspecialist professionals (Q.2.4.5; Multimedia Appendix 2):

We come into contact with older people, who have adult children and I would be asked about grandchildren. So it would maybe, for me even to being a bit more tuned in if someone has got grandchildren.

Interview 02, registered nurse

As measured by the modified SE-12 scale for measuring clinical communication skills, it was identified that participant’s perceptions of self-efficacy to successfully communicate with adults in these situations increased significantly from before (mean 71.25, SE 2.76) to after completion of the e-learning intervention (mean 103.03, SE 1.34; *F*_1,98_=188.059; *P*<.001; Multimedia Appendix 2)*.* Those with previous training reported higher scores of self-efficacy in the pretraining survey, compared with those without training. This difference was statistically significant. However, this gap closed in the posttest survey, where both groups were similar (Multimedia Appendix 2)*.*

In total, 99% (96/97) of participants indicated in the posttest survey that they would be “likely” or “very likely” to apply the learning to their clinical role (Multimedia Appendix 2). Further explored in the qualitative findings, some participants provided examples of when they had used their learning in practice since completing the e-learning intervention. This included professionals encouraging adults that it was in the best interest of the children to tell them the reality of the situation, and providing advice and guidance to adults on how to start the conversation with the children (Q.2.3.3; Multimedia Appendix 2):

And because I had watched that video, I got the confidence to say to him (adult) “you know what, research does actually prove you are better being honest and open.” And he said to me “do you think so?.” I said “yes, honestly trust me’.” And he came back into me on the Monday, and he said “I need to speak to you.” I went oh god it went badly wrong. And he said to me “thank you.” I said “for what?” He said “for giving me that advice, because those children and my daughter and everybody was relieved, and we’ve had the most wonderful weekend.

Interview 12, registered nurse

## Discussion

### Principal Findings

This novel e-learning intervention that is evidence based, theory driven, and free to access provides a much needed and desired educational intervention for professionals to promote end-of-life family-centered cancer conversations [9,38]. The rigorous development of this e-learning intervention was guided by the person-based approach digital intervention development framework [23]. This enabled the incorporation of formative research findings by the team on the support needs of families at the end of life [4,9,39-43], integration of established theories [24,25] with systematic and iterative feedback from end users, and patient and public involvement integrated at all stages of the design and optimization process [26]. Importantly, following engagement with the e-learning intervention, professionals reported an improvement in self-efficacy and enhanced knowledge on the support needs of families at the end of life and felt equipped to have family-centered end-of-life conversations with this population in clinical practice.

The retention rate for the evaluation of this e-learning intervention (99/158, 62.7%) could be considered as positive compared to other nonmandatory, professional e-learning interventions for health and social care professionals [44,45]. Acknowledging the likelihood of a self-motivated population, this study’s high retention rate could otherwise be due to incorporating a range of interactive learning tools, accounting for different learning preferences during the course design. Learning tools comprised flip-card activities, expandable icons, introductory and role-play videos with transcripts, and reflective activities. The role of the learning technologists proved integral [46]; contributing essential skills in learning theory, curriculum development, and course design adapted for the web-based environment [47]. Consequently, pertinent content and design features were integrated for visual and aural preferences (ie, educational video resources) and logical preferences (ie, a step-by-step communication framework [42] and content presented chronologically relative to the end-of-life trajectory) [48]. In addition, intrapersonal preferences were accounted for with reflective activities to give adult learners the opportunity to construct their own knowledge [49]. Other factors that may help explain successful retention rates include the relatively short duration of the e-learning intervention (approximately 40 minutes), avoiding cognitive overloading [50], alongside the careful and respectful use of diverse vector illustrations to moderate for content being considered as lacking cultural relevance [26].

Some professionals stated their motivations for participating in the study was a notable caseload increase of adults at the end of life with significant caregiving responsibilities for children. Within the literature, professionals repeatedly report a desire to provide evidence-based care on how best to support adults to prepare their dependent children for this end of life experience [9,51,52]. As cancer rates increases globally [53], professionals will progressively encounter adult patients with parenting responsibilities who have incurable cancer forming a part of their caseload. It is important for professionals to have readily available educational opportunities to improve self-efficacy and equip them (ie, professionals) with knowledge and tools to engage and progress important family-centered end-of-life cancer conversations [9-11]. This will promote protective opportunities for children to be involved in the end-of-life experience [40]; with educational, social, and psychological benefits, before and after bereavement [3,54]. The advantage of this positively evaluated intervention being available in an e-learning format, in addition to a face-to-face format [14], is the ability to expand, upscale, and disseminate its reach, enhancing global equity [55].

While the posttest survey highlighted a statistically significant increase in scores of self-efficacy, the qualitative data highlighted that professionals would not “shy away from these important conversations” in clinical practice. This is an important finding, in view of the discomfort confronted by professionals caring for families with dependent children at the end of life [9,10]. Consequently, professionals often avoid these challenging conversations; evoked with a sense of helplessness and fear of worsening an emotionally fraught situation [56,57]. However, for most professionals in this study, it was unclear how, or if, skill acquisition to provide family-centered supportive end-of-life care could be inferred from the e-learning intervention. Despite using the most widely cited evaluation framework for educational interventions [29], there remain challenges to determine a causal link between an educational intervention and clinical outcomes. Determining skill acquisition and behavioral application is a well-recognized challenge when evaluating communication skills training [58] and is furthermore acknowledged by our team [14,46]. Of the 4 levels of evaluation within the Kirkpatrick framework (1: reaction; 2: learning; 3: behavior; and 4: results), levels 3 and 4 prove more challenging to report upon, with Kirkpatrick [29] providing limited guidance on how to demonstrate if learning has been applied in practice. Beyond this mixed methods evaluation, there remains a requirement to determine the significance of the training on skill acquisition when professionals are communicating with adults experiencing the end of life, concerning their children [14]. It is therefore considered necessary to evaluate familial impact through the lens of adults and children, to explore if the provision of educational training for professionals has an impact on the care and support received at the end of life.

Of note, the findings highlighted the applicability of the e-learning intervention beyond the intended target audience of adults with cancer who have significant caregiving responsibilities for children; with transferability to those with other close adult-child relational bonds (such as grandparents) and to other life-limiting conditions. Although participants from 14 countries completed the e-learning intervention, it is unclear how applicable and relevant the educational intervention would be for professionals and families from non-Western contexts. However, there are core principles that are important irrespective of cultural context [58]. This includes the following: (1) exploring adults’ beliefs, attitudes, and readiness to share the poor prognosis with the children; (2) explaining to adults the benefits of telling the children about the poor prognosis; and (3) children’s developmental understanding of illness and death [59,60]. There is the potential for adaptation of this e-learning intervention to specific cultural contexts, to further meet the current global gap of family-centered end-of-life educational interventions for professionals [14]. Despite reported barriers, such as scarce technology and internet access, low digital literacy, and poor infrastructure in low-income and middle-income countries [61], there are emerging data that the world’s highest low-income countries are progressing rapidly with regard to implementing e-learning technology [62], closing the digital health care educational divide.

Most professionals highlighted the beneficial learning obtained from viewing, and for some reviewing, the videos within the e-learning intervention that role-played parent-professional communication across the end-of-life trajectory. However, to enable further application of the learning and to problem-solve challenging parent-professional conversations, several participants indicated the desire for experiential and participatory learning in a training environment. Role-play has been advocated as an effective means for professionals to gain confidence in communication skills [63]. Some communication skills training programs offer blended learning with actors or “standardized” patients to help learners gain experience in simulated clinical environments [64]. Nonetheless, the training of the actors or “standardized” patients is costly and time-consuming [65]. Alongside this, individuals who are actors for standardized patient-professional role-play may present biases and experience feelings of anxiety, fatigue, and physical discomfort, albeit with limited long-term negative consequences [66]. With technological advances, there is an opportunity to use artificial intelligence and machine learning to generate natural language within the context of simulated virtual patients, enabling interaction with professional learners for the development of communication skills [67]. The evidence collated from a recent scoping review rated the use of artificial intelligence and machine learning in communication skills training as viable and highly promising [68]. Using digital learning enables the creation of various scenarios that could be customized to specific real-life situations [69], such as adult-professional end-of-life communication. Professionals have the potential to benefit from practicing simulated patient encounters that are tailored to the challenges they confront in routine clinical practice and gain real-time feedback.

### Study Strengths and Limitations

At project commencement, the research team considered collecting engagement and preference data analytics with a desire to gain an understanding of patterns, trends, and interactions with the e-learning intervention, such as the amount of time spent on each section, where individuals dropped off, and if users returned to sections. Taking into consideration the data analytics process with the learning technologists, the content would have required development as “microlearning blocks” on Rise360. This would incur additional development costs, necessitating 5 “microlearning blocks,” alongside the requirement of learners to “scroll and click” at multiple points to progress through sections within the e-learning intervention. Following user consultation, this concept was not pursued to reduce navigational burden and user fatigue. Furthermore, the team took cognizance of the rigorous and rich learning obtained from the iterative user-testing process undertaken in adapting and optimizing the e-learning resource with end users, which provided data on engagement and preferences [26]. In addition, the planned qualitative element of this mixed methods outcome evaluation enabled the collection of usability and acceptability data. Consequently, the e-learning intervention was developed as one sharable content object reference model (SCORM) package.

The e-learning intervention was hosted on the All-Ireland Institute of Hospice and Palliative Care educational hub, being a leading organization with national and international influence and promoting excellence in palliative care. Our team established a collaborative agreement for hosting the e-learning intervention from study inception (ie, grant writing), which proved valuable in promoting the professional integrity of the educational intervention, mitigating platform hosting costs, and enabling longevity of the intervention beyond the study period. A potential drawback of having the e-learning intervention hosted and embedded on an organizational learning platform is professionals who search on Google or Bing (which accounts for 93% of all internet searches [70]) for educational interventions are less likely to identify embedded courses on an initial search, which could impact the reach of the intervention. Platform hosting costs, reach, and infrastructure should be important considerations for funders and researchers of e-learning interventions at project commencement.

### Conclusions

Engagement with this coproduced, theory-driven, and evidence-based e-learning educational intervention demonstrates positive improvements in professionals’ self-efficacy and preparedness to engage in supportive parent-professional end-of-life cancer care conversations, when an adult with significant caregiving responsibilities is dying. Having the educational intervention in a freely available e-learning format has the potential to improve reach at a national and international level, bridging the recognized global gap of educational interventions in this field. Findings indicated applicability of the e-learning intervention beyond the intended target audience of adults with cancer who have significant caregiving responsibilities for children, with transferability to those with other close adult-child relational bonds and to other life-limiting conditions. Equipping professionals on family-centered supportive care has the potential to promote a better end-of-life experience for the family and mediate adverse outcomes before and after bereavement.

## Appendix

Multimedia Appendix 1 Modified Self-Efficacy Questionnaire tool.

Multimedia Appendix 2 FCCCEoLEd e-learning Evaluation Pillar Integration Process with joint display of quantitative (pretest and posttest surveys) and qualitative data.

## Abbreviations

SCORM: sharable content object reference model
SE-12: self-efficacy questionnaire

## Data Availability

The dataset that supports the study findings is available at the University College Dublin Repository and available upon reasonable request from the first (CJS) and second (CON) authors (principal investigators of the study). The data are not publicly available due to ethical restrictions, as detailed in the ethics committee approval from Ulster University (FCNUR-23-002) and University College Dublin (LS-22-65).
