# Supplementary material for: An e-Learning Intervention for Professionals to Promote Family-Centered Cancer Care When a Significant Caregiver for Children Is at End of Life: Mixed Methods Evaluation Study
Source: J Med Internet Res. 2024 Dec 10;26:e65619. doi: 10.2196/65619 (PMC11668990; doi:10.2196/65619)
Supplement: Multimedia Appendix 1 [file jmir_v26i1e65619_app1.docx]

**The following questions relate to how certain you are that you can successfully communicate with adults with incurable cancer concerning their significant caregiving responsibilities for children (<18 years old)**

Please rate your self-efficacy on each of the following 12-items on a scale from 1 – 10

**Scale**

**1     2     3     4      5      6    7    8     9    10**

☐     ☐   ☐   ☐     ☐    ☐   ☐  ☐    ☐   ☐

   Very                                                     Very                                          uncertain                                                certain

**How certain are you that you are able to successfully…**

| identify the issues adults with incurable cancer wish to address during the conversation? | **1     2     3     4      5      6    7    8     9    10**  ☐     ☐   ☐   ☐     ☐    ☐   ☐  ☐    ☐   ☐ |
| --- | --- |
| make an agenda/plan for a conversation with the adults? | **1     2     3     4      5      6    7    8     9    10**  ☐     ☐   ☐   ☐     ☐    ☐   ☐  ☐    ☐   ☐ |
| urge adults to expand on their problems/worries about telling the children the incurable cancer? | **1     2     3     4      5      6    7    8     9    10**  ☐     ☐   ☐   ☐     ☐    ☐   ☐  ☐    ☐   ☐ |
| listen attentively to adults with incurable cancer about their concerns for the children without interrupting or changing of focus? | **1     2     3     4      5      6    7    8     9    10**  ☐     ☐   ☐   ☐     ☐    ☐   ☐  ☐    ☐   ☐ |
| encourage adults with incurable cancer to express thoughts and feelings about telling their children about the prognosis? | **1     2     3     4      5      6    7    8     9    10**  ☐     ☐   ☐   ☐     ☐    ☐   ☐  ☐    ☐   ☐ |
| structure conversations with adults with incurable cancer about their children? | **1     2     3     4      5      6    7    8     9    10**  ☐     ☐   ☐   ☐     ☐    ☐   ☐  ☐    ☐   ☐ |
| demonstrate appropriate non-verbal behaviour (eye contact, facial expression, placement, posture, and voicing) when talking with adults with incurable cancer about their children? | **1     2     3     4      5      6    7    8     9    10**  ☐     ☐   ☐   ☐     ☐    ☐   ☐  ☐    ☐   ☐ |
| show empathy (acknowledge the adult’s views and feelings about telling their children about the incurable cancer)? | **1     2     3     4      5      6    7    8     9    10**  ☐     ☐   ☐   ☐     ☐    ☐   ☐  ☐    ☐   ☐ |
| clarify what adults with incurable cancer know in order to communicate the right amount of information with their children? | **1     2     3     4      5      6    7    8     9    10**  ☐     ☐   ☐   ☐     ☐    ☐   ☐  ☐    ☐   ☐ |
| check adult’s understanding of the information given about an incurable cancer diagnosis? | **1     2     3     4      5      6    7    8     9    10**  ☐     ☐   ☐   ☐     ☐    ☐   ☐  ☐    ☐   ☐ |
| make a plan with adults about how to tell the children about the incurable cancer diagnosis? | **1     2     3     4      5      6    7    8     9    10**  ☐     ☐   ☐   ☐     ☐    ☐   ☐  ☐    ☐   ☐ |
| close the conversation by assuring that the concern and questions of adults with incurable cancer about their children have been answered? | **1     2     3     4      5      6    7    8     9    10**  ☐     ☐   ☐   ☐     ☐    ☐   ☐  ☐    ☐   ☐ |
