# Supplementary material for: An e-Learning Intervention for Professionals to Promote Family-Centered Cancer Care When a Significant Caregiver for Children Is at End of Life: Mixed Methods Evaluation Study
Source: J Med Internet Res. 2024 Dec 10;26:e65619. doi: 10.2196/65619 (PMC11668990; doi:10.2196/65619)
Supplement: Multimedia Appendix 2 [file jmir_v26i1e65619_app2.docx]

**Table 2.** FCCCEoLEd e-learning Evaluation Pillar Integration Process with a joint display of quantitative (pretest and posttest surveys) and qualitative data^a^.

| Quantitative data | Quantitative category interpretation | Pillar building themes | Qualitative category interpretation | Qualitative data |
| --- | --- | --- | --- | --- |
| The e-learning resource:   - Graphics/media were purposeful (mean 4.76, SD 0.495)   The e-learning resource   - Made me feel engaged (mean 4.67, SD 0.703) - Was easy to use (mean 4.74, SD 0.485) - Was clear and organized (mean 4.84, SD 0.373) - Layout was appealing (mean 4.71, SD 0.539) - Language was easy to understand (mean 4.71, SD 0.407) - Was interesting (mean 4.76, SD 00.428) - Was a worthwhile use of my time (mean 4.65, SD 0.613)   If you had a choice of delivery for this training program which would you choose?   - e-Learning—77.3% (n= 75/N) - Face-to-face—22.7% (n=22/N)   The length of the e-learning was   - Too long—1% (n=1/N) - Just right—92.8% (n=90/N) - Too short—6.2% (n=6/N) | - Items relating to the content and layout of the resource were rated highly by participants. | Pillar 1: experience of engaging with the e-learning resource—the qualitative data complemented the quantitative data to describe participants’ positive perceptions of the usability of the resource. Most participants were satisfied with the format of the e-learning intervention. Quantitative data highlighted that most participants preferred the e-learning format compared with face-to-face facilitation. However, in the qualitative data, participants indicated a preference for elements of the training to be offered face-to-face. The qualitative data also enhanced the understanding of what elements of the e-learning intervention enhanced participants’ engagement. | Most participants were positive about the videos that were integrated in the resource and felt that they were helpful to their learning. Two (%) participants did raise some concerns that their videos were brief, highlighting a preference for additional videos on how to navigate complex familial situations.  In terms of the education being a digital resource, this was received positively by participants. Participants found the e-learning intervention easy to use and complemented the flexibility of completing it in their own time. They also found the length of the resource favorable. Some participants felt that they may have benefited from some blended learning elements. In particular, an opportunity to practice their learning from the intervention in a roleplay, in-person environment. Participants had a generally positive experience completing the e-learning intervention. Participants expressed that the content and information provided was clear and well explained. Participants also enjoyed the interactive elements and reflective exercises throughout. | Subtheme 1.1—reaction to the videos:   - Q.1.1.1: “I enjoyed the videos. I think and maybe that’s just me being that type of learner you know, I’ve always enjoyed being able to observe practice. I think that really consolidates anything, you know any written material or any theory, or for a guidance. I think it really gives another insight, a deeper insight to it.” [Interview 10, registered nurse] - Q.1.1.2: “I found the video clips very, very helpful in the e-learning.” [Interview 05, registered nurse] - Q.1.1.3: “It’s only a personal thing, but I found the role-plays a little off putting. Because in some ways they would seem too easy. So I think the information is helpful. I found the role plays less useful. But that’s my own personal reflection.” [Interview 09, palliative medicine consultant] - Q.1.1.4: “More conversations examples would be useful as not all families are smooth-going.” [Open-ended response to posttest, registered nurse]   Subtheme 1.2—look and feel of e-learning resource:   - Q.1.2.1: “It’s fine, easy to follow. Once you were in, once you figured it out, it was easy enough to follow. Most people are used to eLearning nowadays with online.” [Interview 01, registered nurse] - Q.1.2.2: “And it was very good that you could do a wee bit and if you didn’t have time to do the full thing you could come back to where you were and on with another bit.” [Interview 07, registered nurse] - Q.1.2.3: “...would love a follow-up face to face to practice the skill set myself and gain more insight into this topic.” [Open-ended response from posttest, registered nurse] - Q.1.2.4: “...it was a very clear user-friendly way of doing the learning.” [Interview 09, palliative medicine consultant] - Q.1.2.5: “Everything was clearly pointed out very well. And yes, very good presentation as well.” [Interview 05, registered nurse] - Q.1.2.6: “Variation of written, videos and main points kept me engaged throughout course.” [Interview 07, registered nurse] |
| The e-learning resource   - Was relevant to my practice (mean 4.68, SD 0.550) - Will positively impact my practice (mean 4.60, SD 0.589) - Increased my knowledge (mean 4.56, SD 00.677) - Was detailed enough to meet my training needs (mean 4.39, SD 0.848) - Provided appropriate practical examples to support my learning (mean 4.58, SD 0.610)   How likely are you to apply the learning to practice?   - Very unlikely—0% (n/N) - Unlikely—0% (n/N) - Unsure—1% (n/N) - Likely—22.7% (n/N) - Very likely—76.3% (n/N)   Self-efficacy   - Self-efficacy to successfully communicate with adults with incurable cancer concerning their significant caregiving responsibilities for children increased from pretest (mean 71.25, SE 2.76) to posttest (mean 103.03, SE 1.34; n=99; *F*=188.059, *df*=1,98; *P*=<.001) - Test of between subjects on the effect of previous training: *F*=5.415, *df*=1,96; *P*=.022   Before training   - Yes to previous training (m=92.64) - No to previous training (m=69.37)   After training   - Yes to previous training (m=105.64) - No to previous training (m=102.66)   No significant difference of scores of self-efficacy across:   - Years of professional experience as a health care provider (*P*=.554) - Years of experience working in cancer care (*P*=.766) - Having children aged <18 years at home (*P*=.703) - Motivations for undertaking the training (*P*=.728)   What aspect of the resource has been beneficial to your learning (multiple selection options)   - Videos (79/N, %) - Communication framework (57/N, %) - Signposting to other resources (25/N, %) - Reactions according to developmental ages (60/N, %) - Having examples of appropriate language (57/N, %) - Certificate for revalidation (15/N, %)   I would recommend this e-learning resource to a colleague working in cancer or end-of-life care (n=97):   - Strongly disagree—0 (%) - Disagree—0 (%) - Unsure—4.1% (%) - Agree—23.7% (%) - Strongly agree—72.1% (%) | - Participants highly rated items related to the relevancy of the learning, stating it increased knowledge and will impact practice. - There was a statistically significant increase in self-efficacy after completion of the e-learning intervention. - Participants with previous training reported higher scores of self-efficacy in the pretraining survey, compared to those without training. However, this gap closed in the posttest survey, where both groups were similar. This result was statistically significant. | Pillar 2: professionals feeling equipped to have family-centered supportive end-of-life conversations—integration of qualitative and quantitative data converged to offer an enhanced insight relating to the learning from the resource being applied to clinical practice and the intention to use this learning in practice. The qualitative findings offered further insight into what aspects in particular participants perceived to be most impactful in practice. The qualitative data extended our understanding of the applicability of the learning from the resource to other contexts. | - There was an awareness from participants, before completing the e-learning intervention, that it is important to have a conversation with adults regarding the children. However, participants highlighted that it was beneficial to have evidence-based information surrounding the impact of the unprepared child at end of life. There was a sense of not wanting to cause any upset or harm to families who are already navigating a difficult situation. However, having completed the e-learning intervention, participants feel that they have a stronger justification for encouraging such conversations with adults. - Participants were motivated to apply what they had learned to their clinical practice. Participants felt that they had increased awareness, confidence, and knowledge to be able to initiate these conversations with adults. Participants also spoke about some external facilitators and barriers to applying the learning in practice. - After completing the e-learning intervention, participants felt they were no longer going to shy away from these conversations but be actively initiating end-of-life conversations with those with caregiving responsibilities for children. Two (N=, %) participants had the opportunity to put this learning into practice and initiated end-of-life conversations with families they were caring for since completing the e-learning intervention. When communicating with families when a significant adult is at end of life with cancer, understanding the role of the parent in the decision to tell children was highlighted. Particularly, in reference to balancing resistance from families to tell the children the reality of the situation. - Many participants discussed the usefulness and relevance of the learning from the intervention to be applied in other contexts. This includes the suitability of the resource in other countries outside of the United Kingdom and Ireland and other family dynamics. It was highlighted by some participants that the content would be especially useful as an introduction to this topic and for nonpalliative care specialist health and social care professionals. - Participants highlighted the communication strategies that they have used previously and what they will use since completing the e-learning intervention to engage in these conversations. - An important aspect of communicating with families when a significant adult is at end of life was what language is appropriate to use. Participants spoke about using clear and easy-to-understand language to speak to families and children. This was highlighted as an important learning for participants. - Many participants also recommended the e-learning intervention to their colleagues, and some even discussed recommending the e-learning intervention to their managers so that everyone within their team would be encouraged to complete it. | Subtheme 2.1—increased awareness of the importance of telling children:   - Q.2.1.1: “I suppose it was, the evidence to support it was the key. As a nurse you think it’s important to involve children. Whilst recognising parents know their children better. But I think the key thing was you know the impact this could have on children. I think I’ll always come back to the, it’s nearly the benefit of this you know for the children.” [Interview 10, registered nurse] - Q.2.1.2: “You have a bit more education about why you would encourage people to try and involve them (children). The first thing, they’ll (adults) do is say they don’t want to upset them, and they want to try and protect them. But at least now that I have a wee bit more information and evidence, I can say well actually there’s a good reason for doing this.” [Interview 02, registered nurse] - Q.2.1.3: “As professionals we worry a lot about causing even more upset, but then when you get to the end of the situation where the children are aware, and it can be much more controlled, and you can plan things and the time that you have left isn’t wasted. It gives you some choice and a bit of control over things, so I suppose it’s bearing that in mind and trying to make people see that it’s not an awful thing to do, it’s going to be a painful thing to do but there’s a good reason for it.” [Interview 02, registered nurse]   Subtheme 2.2—increased knowledge and confidence not to shy away from having these conversations:   - Q.2.2.1: “Sometimes you feel like in difficult circumstances that you don’t know what to say, sometimes. I do feel that I was able to reassure and comfort her (adult) more so than if I hadn’t had that training.” [Interview 04, health care assistant] - Q.2.2.2: “I know that this is going to help me grow in the job I’m doing.” [Interview 03, registered nurse] - Q.2.2.3: “I would be more equipped to deal with the adults questions that arise” [Interview 07, registered nurse] - Q.2.2.4: “…it’s made me less fearful, because I didn’t think that was in my role. I didn’t think it was my job, but it is, and now I am very confident to actually do it.” [Interview 12, registered nurse] - Q.2.2.5: “I mean we would be fully supported that you know should the need arise, yes that we would put the learning into practice, yes, within our organisation.” [Interview 07, registered nurse] - Q.2.2.6: “…because time as well, it’s very difficult to find the time even if you really want to spend a bit more time to talk to the patient and the family, sometimes you physically just don’t have the time. I think it’s probably the same across the hospitals and the different ward environments. It’s trying to get time really because we are so busy.” [Interview 05, registered nurse]   Subtheme 2.3—not shying away from conversations:   - Q.2.3.1: “Well I think for me I suppose the key take-home message would be not to shy away from it, not to be scared of having these conversations with people and to try and gently encourage them (adults).” [Interview 02, registered nurse] - Q.2.3.2: “My impression was ‘do not be afraid to bring it up.’ And in fact we should be bringing it up. And it did make me reflect on my practice, because it can be very easy to dodge those questions. But I wonder sometimes whether I also veer away from that conversation a little bit. Yea so it did make me reflect on my own practice a little bit as well.” [Interview 09, palliative medicine consultant] - Q.2.3.3: “And because I had watched that video, I got the confidence to say to him (adult) ‘you know what, research does actually prove you are better being honest and open.’ And he said to me ‘do you think so?.’ I said’ yes honestly trust me’. And he came back into me on the Monday, and he said ‘I need to speak to you.’ I went oh god it went badly wrong. And he said to me ‘thank you.’ I said ‘for what?’ He said ‘for giving me that advice, because those children and my daughter and everybody was relieved, and we’ve had the most wonderful weekend.” [Interview 12, registered nurse] - Q.2.3.4: “I do feel it is informed, you know whilst you feel this is the right thing to do. I think on the one hand you think well this is their child, this is this parent’s child and they know children better. And then it’s, the other bit, but this you know.” [Interview 10, registered nurse]   Subtheme 2.4—applicability to other contexts:   - Q.2.4.1: “I believe it’s not, even though it has been developed in Europe it’s not something that is only applicable to the European context. It’s definitely beyond the European Union geographic catchment.” [Interview 06, palliative care coordinator] - Q.2.4.2: “Culturally for myself, I would say that the training is very western Anglo-Saxon focused. And maybe potentially it’s something that could be reviewed to have I think to also target other priority populations.” [Interview 06, palliative care coordinator] - Q.2.4.3: “And I “It’s important as we’re talking as well, it’s not only children, it’s maybe grandchildren.” [Interview 10, registered nurse] - Q.2.4.4: “And “…we come into contact with older people, who have adult children and I would be asked about grandchildren. So it would maybe for me even to being a bit more tuned in if someone has got grandchildren.” [Interview 02, registered nurse] - Q.2.4.5: “I feel this is good for a general nurse to get foundation skills.” [Open-ended response to posttest, registered nurse] - Q.2.4.6: “I suppose whenever we’re doing our programs we’re thinking about palliative care in its wider sense. It’s not just people with a cancer diagnosis. But equally you know equally this you know and whilst it sits with the cancer head on it. It’s across all progressive conditions.” [Interview 10, registered nurse]   Subtheme 2.5—obtaining new communication strategies and tools:   - Q.2.5.1: “I thought it was very clear because again it was something I wasn’t really aware of and certainly the markers that explained you know the why, where, when, that type of thing you know I found that all really useful because although I won’t necessarily always remember it straight off but if I’m in that situation I have the tools here I can go back and remember.” [Interview 02, registered nurse]   Subtheme 2.6—use of language during EoL conversations:   - Q.2.6.1: “So maybe I might not have been as direct you know. I probably might have tried to pussy foot around certain words. So this course has made me realise no, no, you have to say a spade is a spade.” [Interview 03, registered nurse] - Q.2.6.2: “The language again, the language is really important, the language that we use in those moments.” [Interview 01, registered nurse]   Subtheme 2.7—recommending e-learning to others:   - Q.2.7.1: “I would recommend it, I have said to them ‘I have done this course and I said it’s a fantastic course you should do it’. I said ‘it doesn’t take long to do, everything that’s, the different parts to it, they are all very short, the videos are very short, they’re very concise and to the point. And if nothing else I think you should, you know you would learn quite a little bit from it, quite a good bit from it’.” [Interview 07, registered nurse] - Q.2.7.2: “I went to talk with my supervisor. I believe she could share it with the team you know, or with another team. Because I think they would benefit from taking this course.” [Interview 08, registered nurse] |

^a^A total of 99 participants completed the pretest survey and posttest survey on the main outcome measure of self-efficacy, with 97 participants completing the single-item posttest questions.
